# Strategies to Improve Work Attitude and Mental Health of Problem Employees: Focusing on Airline Cabin Crew

**DOI:** 10.3390/ijerph19020768

**Published:** 2022-01-11

**Authors:** Hwayoung Kim, Myoungjin Yu, Sunghyup Sean Hyun

**Affiliations:** School of Tourism, Hanyang University, 222, Wangsimni-ro, Seongdong-gu, Seoul 04763, Korea; khwayoung@gmail.com (H.K.); rosa7767@hanyang.ac.kr (M.Y.)

**Keywords:** problem employee, work attitude, mental health, job performance, airline, cabin crew

## Abstract

This study examines strategies for improving the work attitude and mental health of airlines’ “problem employees”. Based on a review of previous studies, five different handling methods for problem employees were derived: (1) duty assignment according to ability, (2) confidence beliefs, (3) managerial coaching, (4) human understanding, and (5) mentor system. The study hypothesized that these five approaches influence employees’ work attitudes, mental health, and job performance. To verify these hypotheses, empirical data were collected from 200 airline crew members. The analysis found that only three of the “five different handling methods of problem employees” positively influence job attitudes, mental health, and job performance: (1) duty assignment according to ability, (2) confidence beliefs, and (3) mentor system. In contrast, managerial coaching negatively impacted outcome variables. The study also found that the current handling approaches implemented in the industry have positive and negative outcomes on problem employees. Therefore, airline companies need to manage problem staff based on the findings of this study. Particularly, when conducting managerial coaching, supervisors should check employees’ work attitude change status. Research implications, limitations, and future research directions are discussed.

## 1. Introduction

The airline industry is a labor-intensive industry [1]. Specifically, it is a business that requires a great deal of labor to produce goods and services. In particular, the cabin crews as front-line employees are mentally and physically engaged, given that passengers spend most of their time on board [2].

Cabin crew members work in limited space and time, concretely, and they work in an uncommon place where they cannot directly contact the outside world [3]; therefore, teamwork is a crucial factor in sophisticated work performance [4]. In the case of Korean Air, intensive efforts have been made to improve the teamwork of cabin crews to maximize their work efficiency. Teamwork not only affects team performance in a unique working environment but is also directly associated with customer service quality [3]. Given this fact, cabin crew members perform as a team of more than 10 members during a long-range flight, so even a minor mistake by one can negatively affect the remaining team members.

Some cabin crew members have a negative influence on the overall team’s performance due to maladjustment or neglect of work. Researchers have so far defined it as problem employee, or problem staff [5]. In general, these problem employees are classified into various categories, based on poor job performance, lack of cooperation with others, resistance to change, and disobedience to boss feedback. Most problem employees among the cabin crew possess certain characteristics, such as providing passengers with poor service quality, no cooperation with team crew members, lack of self-management, inadequate knowledge regarding work, and failure to respond to supervisors or feedback. Considering that employees with problems in flight work can be assigned to the team, managers are always considering ways to maximize their capabilities.

A major challenge within an airline is handling problem employees in teamwork. While service and technology can be controlled and managed, it is challenging to deal with various people. From the perspective of the airline, it is expected that managers can recognize problem employees on the team, adapt to their work, and increase their abilities to the maximum. In fact, there are previous studies related to teamwork within airlines. Ko, Lee, and Hyun [3] conducted a study to investigate the advantages of the airline team system. Chung and Chang [6] examined whether the self-emotional ability and social-emotional ability of airline crew had a significant effect on teamwork capability in airline services. And Ford, O’Hare, and Henderson [7] conducted research to effectively improve the communication and teamwork of cabin crews during flight operation by applying social categorization and social identity theory. As such, there are studies related to the teamwork of cabin crews in airlines, but there have been no studies analyzing how to manage problem employees that reduce the efficiency of teamwork in airlines.

Therefore, the purpose of this study is as follows: first, to present effective handling methods for problem employees through a literature review, second, to examine the causal relationships among the methods of handling problem employees, work attitudes, mental health, and job performance, and third, to suggest implications to improve the work ability of problem employees based on the analysis results.

## 2. Conceptual Framework

### 2.1. Problem Employee

The term “problem employee” includes comprehensive interpretations, not simply an employee who is creating trouble. The term is used for employees who are indolent, lack ability, and have failed to perform in a project [8,9,10,11]. Accordingly, a “problem employee” is someone who continuously, negatively impacted the company [12,13,14,15,16,17,18,19,20,21,22,23,24,25,26]. Undeniably, all companies have reported “problem employees” [27]. By far, industrial managers’ main concern is how to recognize these “problem employees.”

Unfortunately, there is no way to conclusively identify these types of “problem employees.” This can be attributed to the absence of personal records of individual employees, which can assist the company in identifying a problem employee [28]. Therefore, it would be burdensome for managers to recognize such employees. However, at the worksite, managers tend to avoid or acknowledge working with these problem employees, which might reveal their lack of expertise in handling these crew members in their team.

Consequently, handling “problem employees” could be a challenging task for managers at the workplace [29]. In particular, it can be critical for companies, such as Korean Air, that work as a team. Dereliction of duty on the part of one problem employee can directly affect team performance. Human resource and management studies have discussed how to handle and adequately use these employees for greater benefits in management.

### 2.2. Handling Methods on Problem Employee

#### 2.2.1. Duty Assignment

The most favorable choice that numerous managers assumed to handle these “problem employees” was giving them an appropriate assignment at work based on their capability [30]. Allocating the right assignment to the right person can inspire their ability to bring a positive outcome; moreover, it is a measurable result [31].

“A fitting job assigned to employee’s ability” should be based on thoughtful consideration of understanding individual talent. It is a demanding skill for managers to assign a straightforward but challenging task for problem employees in the long term [30]. In terms of the service industry, this is not an exception. For example, in the airline industry, assigning a less burdensome area to the problem crew is the most practical case as a fitting job assignment. Every person has different talents and capabilities; thus, thoughtful consideration before assigning a task is necessary. The allocation of the right assignment to the right person can reduce the failure to achieve the team’s goal.

#### 2.2.2. Core Confidence

Confidence building is the standard method to help employees handle work/social/human relationships [32]. Confidence refers to the feeling of being able to do something or do it well. Confidence is also defined as the certainty that people are capable of managing whatever is assigned to them or what they intend to do [32].

The opposite of confidence is uncertainty. Specifically, employees do not know how to handle their work. Mittal, Ross, and Tsiros [33] mentioned that confidence is the most important motivation for performing tasks justifiably. Mittal et al. [33] demonstrated that employees tend to assume risks and challenge independently when making decisions with confidence. Prior studies have highlighted the important of instilling confidence in problem employees. Studies have shown that employees who have confidence may have (1) enhanced performance [34], (2) the right attitude toward their duty [35], and (3) a higher workplace happiness index [36].

Finally, confidence drives positive changes in the work attitude, and when performance improves, it results in a higher happiness index [36]. Boosting the confidence of problem employee is an effective way to manage them, while also maintaining a good relationship.

#### 2.2.3. Managerial Coaching

Managerial coaching is a one-on-one discipline that leads to succession so that “the goals desired by the manager” can be achieved [37]. Strict guidance sometimes requires providing coaching to problem employees to develop training, boost job performance, and achieve the desired goals, which is distinct from simply harassing employees [38].

Scholars have used managers’ strict supervision and mentoring independently [39]. The mentor should be a skilled person in the job and present mentees with guidelines for career development and networking [40]. However, under strict guidance, a leader does not necessarily have to be a skilled person because he/she has already handled certain tasks (e.g., duty in flight) and can impart discipline to his/her successor in this regard.

Kim et al. [41] noted that managerial coaching, unlike mentoring, is a short-term map that allows “immediate improvement” to be made during work to ensure that subordinates evolve. Providing constructive advice to problem employees for specific actions can develop and enhance their performance in a work environment [42].

#### 2.2.4. Human Understanding

Managers’ human understanding of employees has received widespread attention in various fields, including leadership and mentoring literature. Managers’ human understanding involves approaching and communicating, being contemplative, being patient, helping, listening to problems or complaints, and considering the needs of employees [43,44]. This human understanding can be considered a type of social support that helps relieve employee stress [45].

Essentially, managers’ human understanding of problem employees is the process of listening, understanding, and helping ease stress related to work or non-work. Human understanding can be achieved by bonding with one another. It is a process of paying attention to problem employees and understanding them thoroughly through continuous conversation. Sometimes it is not easy to understand them because it involves a great deal of emotional consumption, but if managers continue to build exceptional relationships, emotional exchanges will naturally deepen.

#### 2.2.5. Mentoring System

People form relationships with different people in an organizational setup. The special and strong relationships that experienced and competent colleagues form with their colleagues to help them adapt to the organization’s formal or informal norms and solve problems related to the organization’s work are called mentoring [46,47,48,49].

Mentoring can be categorized into formal and informal types [50]. Formal mentoring is established by mentors and mentees in consideration of individual or organizational purposes by their superiors. The duration and scope of the mentoring are designed, and the frequency of learning experience and contact is planned. However, in informal mentoring, mentors and mentees are naturally connected, and the focus of mentoring is on employees, not organizations. The duration and scope of mentoring can be short or long, and the frequency of learning experience and contact may not go as planned, unlike formal mentoring.

Korean Air is implementing mentoring systems to improve the performance of junior cabin crew. To accommodate interns with less than two years of service, each flight team operates the mentoring system through continuous management and evaluation.

### 2.3. Work Attitude

Work attitude refers to the employees’ feelings regarding various factors in the work environment. It also defines positive or negative responses to the work environment [51]. Work attitude refers to a mindset or position taken in response to an external environment or situation. Therefore, heterogeneous attitudes toward duties have a significant impact on the performance of an organization or individual. If an employee has a positive work attitude about his/her job, he/she will be more concerned about the job [52]. Therefore, work attitudes can be closely related to the performance of an organization or individual.

There are various perspectives on the scope of work attitudes, and the most common variables used to measure work attitudes in studies are job satisfaction and organizational immersion [53]. Therefore, this study intends to focus on job satisfaction and organizational immersion.

#### 2.3.1. Job Satisfaction

Job satisfaction is defined as an overall assessment of work [54]. Particularly, job satisfaction is the level of satisfaction with the job [55]. It is an important aspect of the organization because employee satisfaction eventually leads to positive work performance.

In the airline industry, the crew’s job satisfaction has a direct impact on the service attitude toward the customer, which in turn has a positive impact on the airline’s image. In addition, positive work attitudes based on job satisfaction can result in positive feedback from passengers and colleagues, leading to persistent job satisfaction.

#### 2.3.2. Organizational Immersion

Organizational immersion refers to the extent of engagement in an organization. It implies that members of the organization are inclined toward the organization in terms of loyalty, identification, and participation [56]. In addition, the dictionary meaning of immersion is to dig deep into or out of a particular thing, which means immersion within a group of members.

Organizational immersion is a topic that has drawn sufficient attention from scholars who study organizations. The organization has recognized that task immersion is important in managing human resources and has drawn steady attention.

### 2.4. Mental Health

According to the World Health Organization, “health is not just a state of being disease-free, but a state of well-being that is socially, mentally and spiritually satisfied.” While physical health is an important aspect in humans, the importance of mental health has also been recognized as a significant factor, compared to the past, especially in modern society.

The definition of mental health began with a psychopathological concept to refer to the presence or absence of mental illness, but it is now defined as a universal term in various ways to represent a normal individual’s mental state. In addition, mental health is defined as a state of well-being, satisfaction, and mature personality that can maximize the ability to adapt to the environment and handle one’s life independently and constructively [57].

In the airline industry, taking care of mental health is considered an important issue, especially when the number of airline crews is decreasing. Nowadays, mental health is considered an essential component of health that expresses a state of well-being in which individuals can productively work to achieve success and contribute to their communities.

### 2.5. Job Performance

Job performance refers to the degree to which an employee accomplishes a task, and generally refers to the extent to which an employee can achieve a desired state or goal [58]. The job performance of team members is related to organizational productivity. Airlines generally employ the following three methods to measure and evaluate the job performance of the cabin crew.

#### 2.5.1. Employee Assessment

Employee assessment has two functions within an organization. The first is based on decisions within the organization, such as promotion, dismissal, and restructuring of employees. The second helps employees identify and plan opportunities to grow [59].

In the case of several airlines, the average result is known by adding up the assessment received every six months. There are two evaluations: the first is an in-team evaluation by the team leader of the concerned team, and the second is an in-team evaluation by the team leader of another team. The two assessments will be classified into S, A, B, C, and D to evaluate the results of the six-month flight assessment.

#### 2.5.2. Teamwork

Several airline companies fly on a team system owing to their management efficiency. The D airline’s cabin crew consists of teams on a yearly basis and operates about 80 percent of the flights monthly. Teamwork has been defined in diverse ways by many scholars. Lee, Bang, and Shonk [60] called it a group formed to achieve the same goal, while Hatcher and Rose [61] defined teamwork as a group process characterized by cooperation, goodwill, interactive communication, and coordination of group efforts.

Accordingly, teamwork is closely related to the performance of members, so the subjective evaluation among members that teamwork is good or bad is also an aspect that cannot be ignored. Given this composition of teamwork, it is believed that individual team members’ capabilities not only affect the overall team’s performance but also the intimacy and bond between team members.

#### 2.5.3. V.O.C (Voice of the Customer)

The cabin crew also works at customer contact points. Therefore, evaluations, such as complaints and compliments from passengers, can directly affect their work performance. VOC stands for Voice of the Customer, meaning the “sound of the customer.” The customer’s voice regarding the service includes suggestions, appreciation, and dissatisfaction. Airline companies attempt to address various issues by analyzing VOCs.

Some airlines evaluate the job performance of the cabin crew depending on the degree of receiving these VOCs, even though it is the subjective opinion of passengers. Active service recovery activities to resolve VOC complaints can be an objective assessment of the problem employee and is expected to have a significant impact on the airline’s quality of service improvement.

## 3. Research Methods

### 3.1. Research Models and Hypotheses

This research was conducted to clarify the relationship between five handling method variables and lagging variables that enhance the job performance of cabin crew. Based on the theoretical background, this study presents the following eight hypotheses.

**Hypothesis** **1** **(H1).** *Duty assignment based on job ability has a positive (+) influence on work attitude*.

**Hypothesis** **2** **(H2).** *Delivering core confidence brings a positive (+) influence on work attitude*.

**Hypothesis** **3** **(H3).** *Managerial coaching has a positive (+) influence on work attitude*.

**Hypothesis** **4** **(H4).** *Human understanding has a positive (+) influence on work attitudes*.

**Hypothesis** **5** **(H5).** *A mentoring system has a positive (+) influence on work attitude*.

**Hypothesis** **6** **(H6).** *Work attitude has a positive (+) influence on mental health*.

**Hypothesis** **7** **(H7).** *Mental health has a positive (+) influence on job performance*.

**Hypothesis** **8** **(H8).** *Work attitude has a positive (+) influence on job performance*.

**Figure 1:**
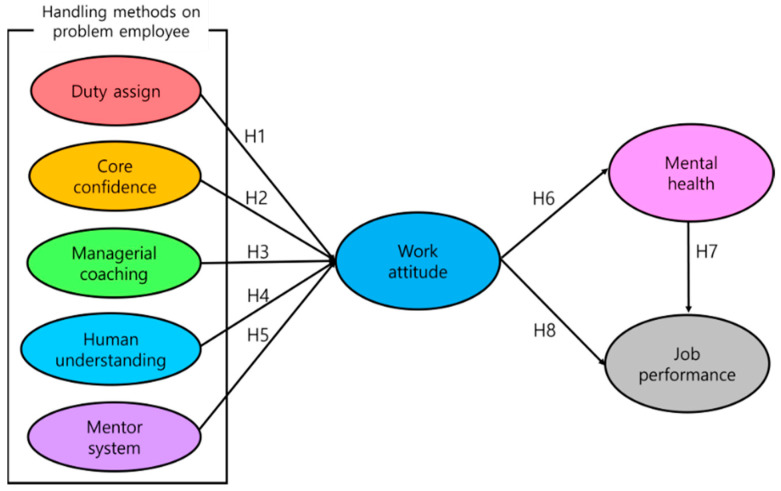
The research model.

The research model was established based on these eight hypotheses. Figure 1 explains the research model and hypotheses.

### 3.2. Collecting Survey Data and Analysis Methods

To validate the measurement items of this study, a preliminary survey was conducted on cabin crew (n = 30) prior to the actual survey. After supplementing the problems raised through the preliminary survey, the Korean Air cabin crew were polled for 90 days from 1 January–31 March 2021. The study subjects included former and current managerial positions of the cabin crew. The questionnaires were collected by delivering online Google surveys to the applicants through SNS and face-to-face. Collecting surveys through SNS was the same as before, but it was difficult to collect surveys through face-to-face at airports because flights were greatly reduced due to the influence of the COVID-19 pandemic. The online surveys were conducted with 200 applicants using a convenient sampling method. All 200 valid samples were used for statistical analysis.

For statistical analysis for hypothesis verification, frequency analysis, reliability analysis, confirmatory factor analysis, correlation analysis, and structural equation analysis were performed using the IBM SPSS 25 statistical package and the AMOS 25 SEM program.

## 4. Research Results

### 4.1. Demographic Characteristics

For this study, a survey was conducted on cabin crew, and all 200 members who faithfully participated in this survey were selected as subjects. Frequency analysis was used to determine the demographic characteristics of the subjects, and the results are presented in Table 1.

The subjects included 29 men (14.5%) and 171 women (85.5%) of which 12 were aged 31–35 (6.0%), 82 were 36–40 (41.0%), 74 were 41–45 (37.0%), and 32 were aged 46 or older (16.0%). The sample included 75 unmarried (37.5%) and 125 married individuals (62.5%). Additionally, 11 (5.5%) had a college degree, 151 (75.5%) a university degree, 24 (12.0%) were in graduate school, and 14 (7.0%) had a graduate degree. The average working period was 17.28 years, with 14 people (7.0%) having less than 10 years, 69 (34.5%) between 11 and 15 years, 72 (36.0%) between 16 and 20 years, and 45 (22.5%) 21 years or more.

There were 16 assistant pursers (8.0%), 138 pursers (69.0%), 40 senior pursers (20.0%), and 6 chief pursers (3.0%). Most were current cabin crew members (145 people; 72.5%), while 55 (27.5%) were former. The annual salary of eight people (4.0%) was between 40 and 49 million won (4.0%), 16 reported (8.0%) between 50 and 59 million won, 71 (35.5%) between 60 and 69 million won, and 105 (52.5%) with 70 million won or more.

### 4.2. Confirmative Factor Analysis and Validation Results

#### 4.2.1. CFA on the Measurement Model and Reliability Results

A confirmatory factor analysis was conducted to validate the impact of the handling methods of problem employees on work attitude, mental health, and job performance.

**Figure 2:**
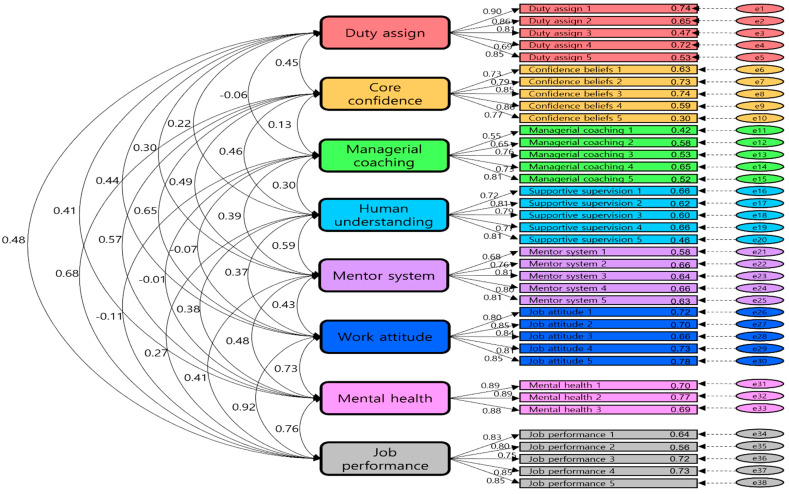
Confirmative Factor Analysis Results.

The CFA results on the measurement model are presented in Table 2 and Figure 2, with factor loadings of all items being higher than 0.50 and considered statistically significant. When CFI is greater than or equal to 0.90, TLI is greater than or equal to 0.90, and RMSEA is lower than or equal to 0.08 [62], the results are considered as meeting the target criteria. Considering that the CFI of this measurement model is equal to 0.922, TLI is equal to 0.914, and RMSEA is 0.58, the fitness of this measurement model was exceptional and could be considered reasonable.

Cronbach’s α was used to analyze the reliability of the results for each factor: duty assignment 0.911, core confidence 0.899, managerial coaching 0.827, human understanding 0.886, mentoring system 0.880, work attitudes 0.916, mental health 0.914, and job performance 0.907. Consequently, Cronbach’s α for all categories was above 0.7, and the reliability of the measurement model was considered effective.

#### 4.2.2. Convergence and Discriminant Validity Results

Before proceeding with the structural model analysis, the correlation between the potential variable and the latent variable that composes the potential variable should be sufficiently high; thus, the confirmation of convergence and discriminant validity is a prerequisite. Convergence validity is defined as the convergence level of the observed variables, while discriminant validity is the independence and a correlation factor between potential variables.

First, convergence feasibility can be determined based on construct reliability (CR) and average variation extracted (AVE), which are presented in Table 3. Generally, convergence feasibility is determined to be acceptable when the CR is greater than 0.70, AVE is greater than 0.50 [63], and the CR and AVE of all variables meet the reference values; thus, the convergence feasibility can be determined to acceptable levels.

Second, the results of identifying the correlation relationship between the potential variables of the measurement model are presented in Table 4. Work attitude, mental health, and job performance did not have a statistically significant correlation with managerial coaching, which is an independent variable, and statistically significant correlation with duty assignment, core confidence, human understanding, and mentoring system. This can be considered to mean that managerial coaching methods such as strict guidance, strong supervision, and pointing out problems are not correlated with improving the work attitude, job performance, and mental health of problem employees. In addition, there was a statistically significant correlation between work attitude, mental health, and job performance, especially between work attitude and job performance.

Therefore, Table 4 shows that the highest correlation was between work attitude and job performance. To demonstrate the discriminative validity, the results are presented in Table 5 by comparing the highest correlation between the merged model and the chi-square value of the original model separating work attitude from the job performance into each factor [63].

The chi-square value of the model, which combines work attitude and job performance as a single factor, was 1110.049, and the degree of freedom was 644. The value of the chi-square varies by 51.039, and the degree of freedom varies by 7, with a threshold of 14.067. Specifically, the difference between the chi-square values of the two models (51.039) was found to be greater than the threshold of 14.067; therefore, the model separating work attitude from job performance can be judged to be a better model.

Consequently, it has been confirmed that a model separating work attitudes from job performance is a better model, so the discriminative validity of the measurement model can be determined to be secured.

### 4.3. Analysis of Structural Model and Hypothesis Validation

#### 4.3.1. Model Configuration

**Figure 3:**
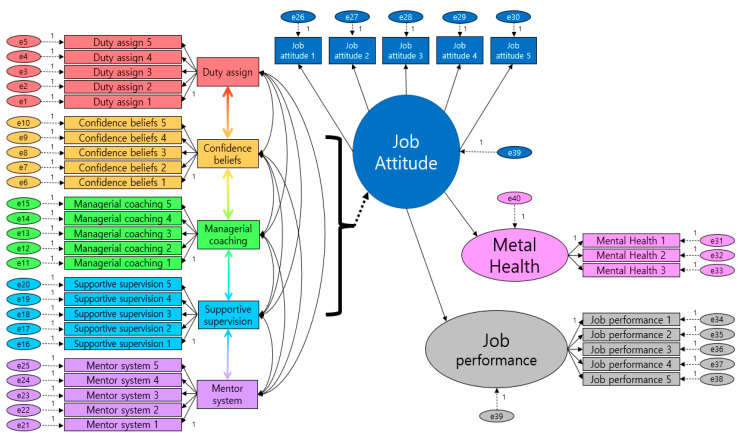
Structural Equation Model.

This study seeks to identify the impact of handling methods of problem employees on work attitudes, mental health, and job performance. Therefore, to achieve the objectives of this study, a structural model was constructed, as shown in Figure 3.

#### 4.3.2. Goodness-of-Fit

To determine the suitability of the structural model constructed for this study, the main goodness-of-fit index was identified, and the results are shown in Table 6.

The major goodness-of-fit index was CFI = 0.918, TLI = 0.911, and RMSEA = 0.059. Both CFI and TLI were above 0.90 and RMSEA was below 0.08, satisfying the goodness-of-fit index reference for the structural model. Consequently, the degree of fit for this structural model was determined to be acceptable.

#### 4.3.3. Structural Equation Model

**Figure 4:**
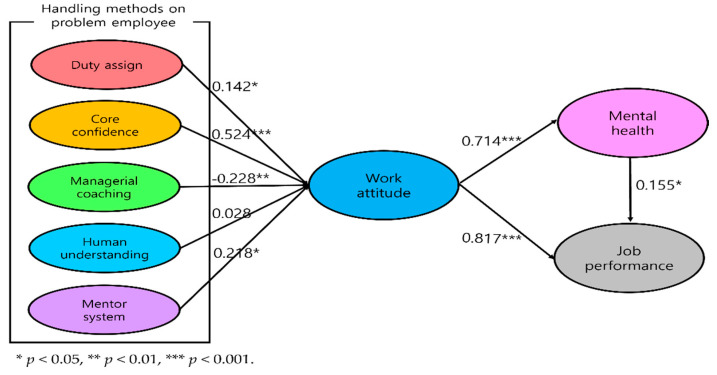
Structural Equation Model Analysis.

To verify the effectiveness of the latent variables in a structural model, the path coefficients in the structural model and their statistical significance were determined, and the results are shown in Figure 4.

Duty assignment based on personal job ability, which was Hypothesis 1, resulted in a significant positive effect on work attitude (β = 0.142, *p* < 0.05). Therefore, H1 was supported. Core confidence, which was Hypothesis 2, had a significant positive impact on work attitudes (β = 0.524, *p* < 0.001). Therefore, H2 was also supported. Managerial coaching, which was Hypothesis 3, resulted in a significant negative effect on work attitude (β = −0.228, *p* < 0.01). Accordingly, H3 was not supported. Human understanding, which was Hypothesis 4, had no significant effect on work attitude. Correspondingly, H4 was rejected. The mentoring system, which was Hypothesis 5, had a significant positive effect on work attitude (β = 0.218, *p* < 0.05). Therefore, H5 was supported.

Work attitude, which was Hypothesis 6, resulted in a significant positive effect on mental health (β = 0.714, *p* < 0.001). Consequently, H6 was supported. Mental health, which was Hypothesis 7, resulted in a significant positive effect on job performance (β = 0.155, *p* < 0.05). Accordingly, H7 was also supported. Work attitude, which was Hypothesis 8, showed a significant positive effect on job performance (β = 0.817, *p* < 0.001). Thereby, H8 was also supported.

## 5. Conclusions

This study sought to analyze the impact of the handling methods of problem employees on work attitude, mental health, and job performance. According to previous studies, five handling methods for problem employees (ability-fitting duty assignment, confidence-providing, managerial coaching through strict guidance, deep understanding through dialog, and a mentoring system) were presented, and the factors had an impact on mental health and job performance through work attitude. The survey was distributed to the cabin crew of Korean Air for 90 days from 1 January–31 March 2021. The study analyzed the responses of 200 cabin crew members who worked as managers in the past or present. Based on the research hypothesis presented earlier, the results of this study are as follows:

First, Hypothesis 1 acknowledged that duty assignments that fit job ability would have a positive impact on work attitude (β = 0.142, *p* < 0.05). Previous studies have mentioned that employees’ job adequacy affects their work attitude [64]. This study extends the scope of prior studies by applying existing findings to the airline industry and empirically verifying them. The airline’s team leader and deputy team leader need a countermeasure to consider problem personnel first in the allocation of duty. The assignment of zones or actual duty assignments can be accomplished without much pressure from problem employees, and assigning positions with less friction with other cabin crews can be a good example.

Second, Hypothesis 2 acknowledged that core confidence would have a positive effect on work attitude (β = 0.524, *p* < 0.001). According to a study by Locke and Latham [32], confidence enhances a positive commitment to achieving goals and promotes positive performance to achieve them. Therefore, managers will be capable of positively changing their work attitude by boosting confidence in various ways whenever problem employees are passive in performing their jobs.

Third, managerial coaching resulted in a significant, but negative effect on work attitude. Therefore, Hypothesis 3 was not accepted (β = −0.228, *p* < 0.01). Given that studies have shown that strict coaching can negatively impact problem employees, this handling method should generally be avoided. Therefore, it would be effective for flight managers to handle problem employees positively in the future rather than providing strict coaching.

Fourth, Hypothesis 4, stating that human understanding of problem employees (dialog, understanding, forming relationships) would have a positive impact on work attitude, had no significant effect on work attitude.

Fifth, Hypothesis 5 acknowledged that the mentoring system has a significant positive effect on work attitude (β = 0.218, *p* < 0.05). This is consistent with prior findings that mentoring systems have a positive effect on work attitude. The advantages of mentoring in an organization/industry have already been discussed through numerous studies [48]. However, research on South Korean airline-related mentoring system has been limited since 2013. In addition, because of the mentoring system for new employees in this study, the results can be considered to be an extended scope of previous studies conducted on existing new employees.

Sixth, Hypothesis 6 acknowledged that work attitude would have a significant positive effect on mental health (β = 0.741, *p* < 0.001). A prior study [65] confirmed that job insecurity negatively impacts mental health owing to stress. Conversely, a positive work attitude can have a positive potential to reach out to stronger mental health, and these results are similar to those of this study.

Seventh, Hypothesis 7 acknowledged that mental health would have a significant positive effect on job performance (β = 0.155, *p* < 0.05). This study is considered an early study that discusses the relationship between mental health and job performance.

In addition, Hypothesis 8 indicated that work attitude would have a significant positive effect on job performance (β = 0.817, *p* < 0.001).

## 6. Implications

The contributions of this study in theoretical and practical terms are as follows:

First, this study is significant in that it directly applies general research on handling problem employees to airline cabin crew to confirm overall efficiency, including job performance, and identify the causality between variables. In addition, the study has presented specific measures and future directions for handling problem employees, which will serve as an opportunity for the airline to derive practical guidelines.

Second, this study differs from other research results in that it vividly captures voices and difficulties through a survey method. Therefore, it was found that the better the duty assignment, the greater the confidence, and the better the mentoring, the higher the work attitude level. However, in the case of the managerial coaching method, it is shown to negatively affect work attitude, so it was possible to break the stereotype of some managers who were obsessed with improving performance in a short period through strict coaching.

Third, this study found that a better work attitude is strictly related to good mental health, resulting in better job performance. When mental health is seen as a social trend issue, the importance of mental health has been realized through this study that it is a direct and decisive factor in the overall improvement of airline work and handling problem employees.

Combining the aforementioned implications and the direction of the findings, airlines should approach each individual considering the usefulness and association of handling measures based on the results shown in this study when managing problem employees. That is, to avoid a uniform approach to problem employees, providing a specific method for each problem employee’s circumstances would effectively enable personnel management and, in the longer term, promote the continuous development of the airline.

## 7. Limitations and Recommendation for Future Research

While the implications of this study have a positive impact on cabin crew management in the future, there are certain limitations owing to the quantitative and qualitative constraints imposed by the survey. Therefore, the direction of future studies is presented as follows, through the confirmation of the study’s limitations.

First, the questionnaire used in this study is self-invasive and relies on subjective perception, and thus cannot guarantee objectivity, fairness, and even anti-dependence on the survey results. In addition, in research dealing with psychological concepts, quantitative research had limitations in deriving a deeper understanding and interpretation of the content under restricted conditions. Therefore, to complement the limitations of self-invasive survey methods, future studies should also adopt in-depth research methods such as individual interviews and participant observation methods for more objective and reliable research.

Second, in deriving the results of this study, it is necessary to recognize the limitations that the actual sample range of the survey is not very wide. Although there have been various airlines in Korea, including low-cost airlines in size and region since the 2010s, it has been pointed out that they have failed to contain various voices. If so, future research should result in handling measures depending on the size or nature of each airline by expanding the scope of the study to other domestic or foreign airlines.

Third, the fact that the negative effect on strict coaching methods was empirically verified throughout the surveys is significant in that it broke existing biases. However, empirically, the positive effects of strict coaching cannot be ignored. Therefore, it is necessary not to uniformly exclude strict coaching methods, but to accurately identify the advantages and disadvantages of strict coaching methods and to suggest ways to significantly compensate for their disadvantages.

As we have seen earlier, for airlines with very high human resource dependencies, the efficiency of human resource management cannot be emphasized more than for any organization. The crew-specific team system is the key to enhancing organizational flexibility and ensuring continuous competitiveness by responding quickly and flexibly to adapt to the rapidly changing work environments. In addition, the impact of individual/subjective factors, such as individual mental health, on individual cabin crew members in a complex and modern society is increasing. Therefore, the strategic management of problem employees among the crew is increasing, and a detailed handling strategy for problem employees must be established where required in the long term.

## Figures and Tables

**Table 1:** Demographic characteristics.

Variables	Index	Frequency (n)	Percent (%)	Mean (SD)
Gender	Male	29	14.5	
	Female	171	85.5	
Age	31–35	12	6.0	
	36–40	82	41.0	
	41–45	74	37.0	
	over 46	32	16.0	
Marital status	Single	75	37.5	
	Married	125	62.5	
Education	College degree	11	5.5	
	University degree	151	75.5	
	In graduate school	24	12.0	
	Graduate degree	14	7.0	
Working period	Less than 10 years	14	7.0	17.28 (4.75)
	11–15 years	69	34.5	
	16–20 years	72	36.0	
	Over 20 years	45	22.5	
Working grade	Assistant Purser	16	8.0	
	Purser	138	69.0	
	Senior Purser	40	20.0	
	Chief Purser	6	3.0	
Working status	Current	145	72.5	
	Former	55	27.5	
Annual income	40–49 million won	8	4.0	
	50–59 million won	16	8.0	
	60–69 million won	71	35.5	
	Over 70 million won	105	52.5	
Total		200	100.0	

**Table 2:** Results of CFA and Reliability Analysis.

Factor	Question	M (SD)	Loading	Cronbach’s α
Duty assignment according to ability	I’ve been trying to assign an easy task to reduce the problem employee’s mistakes.	3.69 (0.91)	0.900	0.911
	I tried to assign an easy zone to the problem employee.	3.59 (0.93)	0.859	
	I tried to take on a new job after the problem employee was perfectly adapted to the current job.	3.67 (0.93)	0.807	
	I didn’t let the difficult task be performed by the problem employee alone.	3.91 (0.84)	0.686	
	I tried to assign a relatively low-intensity task to the problem employee.	3.51 (0.89)	0.847	
Core confidence	I tried to compliment the problem employee for the work he did.	3.94 (0.81)	0.727	0.899
	I helped the problem employee produce new ideas or opinions.	3.70 (0.92)	0.791	
	I made the problem employee proud of his/her job.	3.67 (0.90)	0.854	
	I instilled confidence that I could do anything with the problem employee.	3.79 (0.91)	0.858	
	I found what the problem employee wanted and helped him achieve it.	3.62 (0.87)	0.768	
Managerial coaching	I think I was very strict to properly manage the problem employee.	2.94 (1.01)	0.548	0.827
	I frequently pointed out the problem and offered objective feedback so that the employee did not repeat the mistake.	3.26 (0.96)	0.650	
	I tried to provide the problem employee a strong vision for the job.	3.28 (0.82)	0.760	
	When the performance of the problem employee did not meet the required standards, I encouraged the employee to concentrate.	3.55 (0.83)	0.729	
	I pointed out objectively and clearly when the problem employee did something wrong.	3.64 (0.87)	0.808	
Human understanding	I tried to treat the problem employee humanely at the overseas time.	3.71 (0.89)	0.718	0.886
	I tried to treat problem employees with integrity and honesty.	3.76 (0.84)	0.813	
	I tried to understand the problem employee’s feelings well and showed interest in the problem employee’s point of view.	3.65 (0.83)	0.78	
	I expressed interest in and sympathized with the personal concerns and counseling of the problem employee.	3.67 (0.83)	0.774	
	I tried to share the difficulties experienced by the problem employee.	3.76 (0.80)	0.813	
Mentoring System	I think I tried to assign a good mentor to the problem employee.	3.92 (0.79)	0.680	0.880
	The mentor taught the problem employee the role of the cabin crew, interpersonal skills, and common sense of aviation.	3.78 (0.74)	0.765	
	Mentors actively advised the problem employees about their concerns and mistakes in the workplace and sought solutions together.	3.72 (0.76)	0.810	
	The mentor taught the problem employees special methods or know-how that could help them perform their tasks easily and effectively.	3.83 (0.73)	0.802	
	Mentors helped problem employees perform difficult tasks.	3.81 (0.73)	0.813	
Work attitude	While working in the same team for a year, the job attitude of the problem employee seems to have changed a lot positively.	3.72 (0.80)	0.796	0.916
	While working in the same team for a year, the problem employee seems to have changed to perfectly acquire the working knowledge required for specific tasks.	3.57 (0.82)	0.850	
	While working in the same team for a year, the problem employee tried to use his/her abilities to the fullest to achieve the team’s goals.	3.62 (0.85)	0.840	
	After working for the same team for a year, the problem employee was gradually satisfied with his/her job.	3.62 (0.88)	0.810	
	While working in the same team for a year, it seems that the work attitude of the problem employee had changed positively because it suited his/her aptitude.	3.61 (0.85)	0.853	
Mental Health	The problem employee seemed to have eased a lot of nervousness and anxiety over the past year.	3.59 (0.78)	0.885	0.914
	The problem employee seemed to be more interested in the job over time.	3.57 (0.87)	0.890	
	The problem employee seemed to be a little more optimistic about the future with passing time.	3.67 (0.86)	0.880	
Job performance	While working together for a year, the work ability of the problem employee seemed to have improved immensely.	3.75 (0.81)	0.832	0.907
	While working in the same team for a year, the relationship with the problem employee seems to have changed to become cordial and to work well together.	3.72 (0.74)	0.801	
	While working together for a year, the problem employee seems to have reduced the number of complaints received from passengers or increased the number of compliments received.	3.38 (0.94)	0.749	
	While working for a year, the problem employee seems to have increased the grade of evaluation received by the team leader or other team leaders.	3.43 (0.92)	0.849	
	While flying for a year, the problem employee seems to have changed to provide a service for the convenience from the passenger’s point of view.	3.57 (0.84)	0.852	
	X^2^ = 1059.010, df = 637, *p* < 0.001, CFI = 0.922, TLI = 0.914, RMSEA = 0.058			

**Table 3:** Convergence Feasibility Validation.

Variations	Composite Reliability (CR)	Average Variance Extracted (AVE)
Duty assignment	0.929	0.727
Core confidence	0.921	0.701
Managerial coaching	0.934	0.742
Human understanding	0.918	0.690
Mentoring System	0.930	0.727
Work attitude	0.940	0.759
Mental Health	0.939	0.837
Job Performance	0.932	0.733

**Table 4:** Verification of correlation relationship.

Variations	1	2	3	4	5	6	7	8
1. Duty assignment	1							
2. Core confidence	0.449 ***	1						
3. Managerial coaching	−0.055	0.126	1					
4. Human understanding	0.216 **	0.463 ***	0.299 *	1				
5. Mentoring System	0.297 ***	0.490 ***	0.388 ***	0.586 ***	1			
6. Work attitude	0.435 ***	0.655 ***	−0.066	0.374 ***	0.430 ***	1		
7. Mental Health	0.410 ***	0.572 ***	−0.011	0.383 ***	0.483 ***	0.726 ***	1	
8. Job Performance	0.480 ***	0.679 ***	−0.113	0.269 **	0.413 ***	0.922 ***	0.761 ***	1

Note: diagonal: (normalized), diagonal down: correlation coefficient. * *p* < 0.05, ** *p* < 0.01, *** *p* < 0.001.

**Table 5:** Discriminative validity reasoning based on Bagozzi and Yi.

Model	Χ2	df	ΔΧ2	Δdf	CFI	TLI	RMSEA
Original	1059.010	637			0.922	0.914	0.058
Merge	1110.049	644	51.039	7	0.914	0.906	0.060

**Table 6:** The structural model fit.

X2	Df	*p*	CFI	TLI	RMSEA
1090.177	647	<0.001	0.918	0.911	0.059
